# An Alumina Toughened Zirconia Composite for Dental Implant Application: *In Vivo* Animal Results

**DOI:** 10.1155/2015/157360

**Published:** 2015-04-06

**Authors:** Gianmario Schierano, Federico Mussano, Maria Giulia Faga, Giulio Menicucci, Carlo Manzella, Cristian Sabione, Tullio Genova, Mitzy Mauthe von Degerfeld, Bruno Peirone, Adele Cassenti, Paola Cassoni, Stefano Carossa

**Affiliations:** ^1^Department of Surgical Sciences, CIR Dental School, University of Turin, Via Nizza 230, 10126 Turin, Italy; ^2^CNR-IMAMOTER, Strada delle Cacce 73, 10135 Turin, Italy; ^3^CNR-IMAMOTER (Headquarters), Via Canal Bianco 28, 44124 Ferrara, Italy; ^4^Department of Veterinary Sciences, University of Turin, Largo Paolo Braccini 2, 10095 Grugliasco, Torino, Italy; ^5^Department of Medicine, University of Turin, Via Santena 7, 10126 Turin, Italy

## Abstract

Ceramic materials are widely used for biomedical applications because of their remarkable biological and mechanical properties. Composites made of alumina and zirconia are particularly interesting owing to their higher toughness with respect to the monolithic materials. On this basis, the present study is focused on the *in vivo* behavior of alumina toughened zirconia (ATZ) dental implants treated with a hydrothermal process. A minipig model was implemented to assess the bone healing through histology and mRNA expression at different time points (8, 14, 28, and 56 days). The novel ATZ implant was compared to a titanium clinical standard. The implants were analyzed in terms of microstructure and surface roughness before *in vivo* tests. The most interesting result deals with a statistically significant higher digital histology index for ATZ implants with respect to titanium standard at 56 days, which is an unprecedented finding, to the authors' knowledge. Even if further investigations are needed before proposing the clinical use in humans, the tested material proved to be a promising candidate among the possible ceramic dental implants.

## 1. Introduction

Titanium implants have the longest traceable record of predictable clinical performance with very high success rate [1]; however they are not without possible drawbacks [2]. In fact, titanium might be an allergen [3–5] and may diffuse not only within the adjacent tissues, as it is proven by the elevated concentrations found in the vicinity of oral implants [6] and in regional lymph nodes [7], but also systemically [8]. As a possible alternative to titanium, ceramic materials have been already investigated and clinically used for years.

Alumina and yttria stabilized zirconia (Y-TZP) ceramics are suitable for biomedical applications, due to their good mechanical and tribological properties and proved biocompatibility [9–14]. Pure alumina has been widely used as femoral heads because of its high wear resistance, but no application requiring osseointegration has been implemented to date due to its high inertness. As far as TZP is concerned, it was initially employed to replace alumina in femoral heads owing to its higher fracture toughness, but the high number of failures led to reconsider its suitability in this field. Indeed, depending on temperature, zirconia exists in three phases: monocline, cubic, and tetragonal. Monocline phase is the most stable at room temperature, even if its mechanical properties are inferior to those of tetragonal phase, so that the latter is preferred. However, tetragonal phase should be stabilized to prevent tetragonal phase transformation [15]. Once the transformation occurs, the process continuously proceeds from the surface to the bulk, resulting in a volumetric expansion followed by failure [15]. Even if zirconia materials transform most rapidly at temperature ranging between 200°C and 300°C, at low temperatures the process is enhanced by the presence of water, available* in vivo* [15–22]. Zirconia stabilized materials are employed in orthopedics and dentistry, although in very low percentage with respect to commercially pure titanium and titanium alloys (Ti6Al4V), probably because of the unlucky history as femoral heads [23] and the survival rate inferior to that of titanium implants [24].

To overcome the limits of both monolithic materials, researchers have focused on the preparation of composites made of alumina and zirconia [25–27], already successfully applied as femoral heads [28, 29]. The advantages of these oxidic composites owed mainly to the limited transition from the tetragonal phase to the monocline one, which enhances the mechanical performance [23], and to an increase of the material toughness [30, 31]. The combination of alumina and zirconia allows compensating the moderate toughness of alumina and the ageing effect of zirconia. It has been shown that, when the percentage of ZrO_2_ is kept under the 22% wt [32], ageing phenomena do not occur independently from the grain size.

The mechanical stability is not the only requirement a good implant material should possess, since bioactivity is necessary to ensure proper osseointegration. When dealing with bone bonding materials, bioactivity can be described as the ability to grow bonelike apatite on the material surfaces [33]. Apatite formation in simulated body fluid (SBF) is preferentially induced whenever particular hydroxyl sites are on the surface, which can be achieved through acidic and/or alkali treatments [34]. Consistently, Faga et al. [35] described the formation of acicular hydroxyapatite crystals onto the surface of alumina toughened zirconia (ATZ) samples treated hydrothermally. Furthermore, alumina zirconia ceramics may elicit slightly better biological responses than the commercially pure titanium usually employed for dental implants [36]. Ceramic materials are also very suitable for aesthetic oral rehabilitations, which may be required when dental implants are located in the anterior part of the mouth, as it would preclude the dark shimmer of titanium implants [37–39].

The aforementioned promising properties of ATZ treated with phosphoric acids, in terms of mechanical and bioactivity features, prompted the authors to study the* in vivo* behaviour of such material. For this purpose, implants made of ATZ were placed within the bone of recipient animals, using a titanium clinical standard for comparison since no papers on this topic are present in literature. For sake of completeness, fatigue tests were performed according to UNI EN ISO 14801:2008 standards and surface properties were studied. Indeed, as load bearing medical devices, dental implants are not suitable to clinical use if they present defects possibly affecting their mechanical strength, which may lead to early failure under the chewing load; such might be the case of large porosities within ceramic bulk materials.

## 2. Material and Methods

### 2.1. Implants

Powders with high purity were used to produce the oxidic implants (Tosoh ZrO_2_-20wt %Al_2_O_3_, (TZ-3Y20AB), as “ready to press” powders, so that no additional mixing was required before pressing. Green samples were obtained by linear pressuring at 80 MPa followed by Cold Isostatic Pressing under 200 MPa. The optimized conditions for sintering process were: heating 50°C/h up to 700°C, dwelling for 2 h at 700°C, and heating of 100°C/h up to temperature sintering of 1500°C and dwelling for 2 h at this temperature. Hardness, toughness, and strength of the full dense material was measured on proper specimens, as reported elsewhere by Faga et al. [35]. The materials were then subjected to Computer Aided Manufacturing obtaining one-piece dental implants of 11.5 × 4.25 mm. The surface treatment was obtained by hydrothermal cycles (patent number: TO2012A000029 and PCT/IB2013/050425). Implants were then treated with phosphoric acid under hydrothermal conditions with the purpose of inducing bioactivity [35]. As a control, dental implants with TiUnite surface were purchased from Nobel Biocare (Nobel Biocare Italia, Agrate Brianza, Italy).

### 2.2. Microscopy

Microstructure was studied by means of a Scanning Electron Microscope Zeiss EVO 50 with Energy Dispersion Spectroscopy analyzer for elemental composition detection.

### 2.3. Roughness

The surface roughness was measured by using a noncontact profilometer, Talysurf CCI 3000A on the screw. The tests were performed within an air-conditioned laboratory, where temperature is kept at 20°C, on a representative surface of 90 mm^2^.

### 2.4. Mechanical Tests

Mechanical tests were run in triplicate according to UNI EN ISO 14801:2008 by using a monoaxial machine for both the dynamic and static tests (Italsigma, Italy) equipped with a loading cell (max. load: 3 kN). In static conditions, the speed was 0.2 mm/minute, the preload: 2N. Based on the ISO standards mentioned above, the static tests aim to define the load to be applied when performing the dynamic tests. Therefore, a value inferior to the 80% of *F*_mean_, which is the mean value of the static test strength (*F*_*Max*⁡_) of the three samples, was applied to the samples for 5 × 10^6^ cycles in a sinusoidal way. The minimal strength *F*_min⁡_ corresponded to the 10% of the maximal strength *F*_max⁡_ (*R* = 0,1).

### 2.5. *In Vivo* Experiments

#### 2.5.1. Experimental Design

The* in vivo* experiment was conducted on 16 minipigs. Eight experimental implants per animal were inserted in the right tibia: 4 hydrothermally treated ATZ and 4 Nobel Ti-Unite (Nobel Biocare Italia, Agrate Brianza, Italy). Four animals were sacrificed at 8, 14, 28, and 56 days after the implant placement. The tibias were block-sectioned and subjected to histomorphometric (4 samples per animal) and biomolecular analysis (4 samples per animal). Outcomes were analyzed in terms of new bone apposition by a digital histology index (DHI) and RNA profiling.

#### 2.5.2. Animal

Sixteen adult minipigs (mean weight 65.94 kg SD = 2.84) were used in the experiment (CISRA, Turin, Italy). The minipigs were fed standard pelleted cereal food and were given water* ad libitum*. The animals underwent an acclimation period of 1 week prior to surgery. Abiding with Italian law, all animal experiments were approved by an academic ethics committee.

#### 2.5.3. Surgical Procedure

After preanesthetic sedation with 2% xylazine (Rompun 2%, Bayer, Milan, Italy; 2.3 mg/kg) and tiletamine/zolazepam (Zoletil 100-Virbac 20%, Laboratoires Virbac, Carros, France; 6.3 mg/kg), surgery was performed under intubation anesthesia with isoflurane/halothane and O_2_. The right hind leg was prepared in a standard sterile fashion. After exposing the tibia, the implants were inserted with a 40 Ncm torque. Then, the flap was closed and the surgical access sutured so as to completely cover the implants, whose head reached the bone level. Each tibia received 8 implants: 4 ceramic ones and 4 titanium implants. Tibial bone specimens were collected at this stage to determine baseline (time 0) values for the RNA analysis. At the established time points, animals were euthanized by preanesthesia with 2% xylazine (Rompun 2%, Bayer) (2.2 mg/kg) and tiletamine/zolazepam (6.6 mg/kg) and an intracardiac injection of embutramide, mebezonium iodide, and tetracaine hydrochloride (70 mg/kg). Finally, the tibias were exposed and dissectedinto slices.

#### 2.5.4. Expression of Osteogenic Markers

To protect the RNA, all specimens (8, 14, 28, and 56 days) were placed in RNA Later (Qiagen, Milan, Italy) and stored at −80°C until testing. Before the RNA purification (RNeasy Mini Kit Quiagen, Valencia, CA, USA), the samples were disrupted using a TissueRuptor. Total RNA was subjected to reverse transcription (High Capacity cDNA RT Kit; Cat#: 4368814 Applied Biosystems, USA). The cDNA obtained underwent real-time polymerase chain reaction (RT-PCR) using commercially available primer/probe cocktails for* Sus scrofa* (TaqMan Gene Expression Assays collagen, type I, alpha 1 assay ID: Ss03373340_m1; secreted protein, acidic, cysteine-rich (osteonectin) assay ID: Ss03392006_m1; bone gamma-carboxyglutamate (gla) protein assay ID: Ss03373655_s1; bone morphogenetic protein 2 assay ID: Ss03373798_g1 Applied Biosystems, USA), following the manufacturer's protocol. Glyceraldehyde 3-dehydrogenase (TaqMan Gene Expression Assays, glyceraldehyde-3-phosphate dehydrogenase assay ID: Ss03374854_g1Applied Biosystems, USA) was used as internal control.

#### 2.5.5. Histological Analysis of the Peri-Implant Bone

To evaluate the bone healing and remodeling, histologic analysis was performed at 8, 14, 28, and 56 days after implant. After block section of the implants along with the adjacent bone, the specimens were fixed in 4% formalin for 24 hours and decalcified for 3 to 4 weeks in a mixture of 50% formic acid and 10% sodium citrate tribasic. While the implants were removed, the peri-implant bone samples were embedded with paraffin wax and cut into 3 *μ*m thick sections, along the longitudinal implant axis, using a motorized microtome. Polylysine coated slides were used to enhance the adhesion of the tissue section during staining procedures. The histological structure of the peri-implant bone was assessed by traditional haematoxylin and eosin staining and for optical microscopy. The digital histology index (DHI) was manually determined using an imaging computer software (Olympus Dot Slide BX51) on the virtual histology slide. Briefly, on each virtual slide, the newly formed bone was measured by tracing a line at the interface between bone and implant within a given length (a standard length of 4 cm was adopted). The ratio of newly formed bone to the total bone-implant interface taken into consideration was expressed as a percentage. The DHI was measured on both sides of each slide. Two specimens per time points per material were obtained and at least 10 slides were made from each block sectioned histological sample. In addition, morphometric parameters such as (1) presence of necrotic or fibrous tissue and (2) amount of organized grouped osteoclasts and osteoblasts, together with (3) blood vessels and (4)* de novo* formed bone, were assessed by two independent histologists.

#### 2.5.6. Histomorphometrical Reconstruction

Forty serial sections, 3 *μ*m thick, were cut from the 56-day-paraffin-embedded blocks and stained as described above. Afterwards, the slides were acquired by an automated microscope to generate virtual colored slides, which were converted into grey scale images so as to allow further image processing. The three-dimensional reconstruction of the samples was performed using* Amira 4.0*, an advanced volume modeling software (TGS Template Graphics Software, http://www.tgs.com). Three different volumes were delimitated from the cortex to the medullar space and a percentage of bone density was calculated based on a densitometric analysis of the white voxels, the cortical bone being taken as a reference (100%).

#### 2.5.7. Statistical Analysis

Data from RT-PCR and DHI were analysed by GraphPad Prism 5 (GraphPad Software Inc, La Jolla, CA, USA). RT-PCR was independently repeated at least three times (*n* = 3), on the samples derived from peri-implant bone (*n* = 2 per material per time point). As for the DHI, each specimen (*n* = 2 per material per time point) generated at least 10 virtual histological slides. Statistical analysis was performed by using the one-way analysis of variance (ANOVA) with* post hoc* Dunnett's test or the Student's *t*-test, as appropriate. A *P* value of <0.001 was considered significant.

## 3. Results

### 3.1. Morphology and Surface Analysis

**Figure 1:**
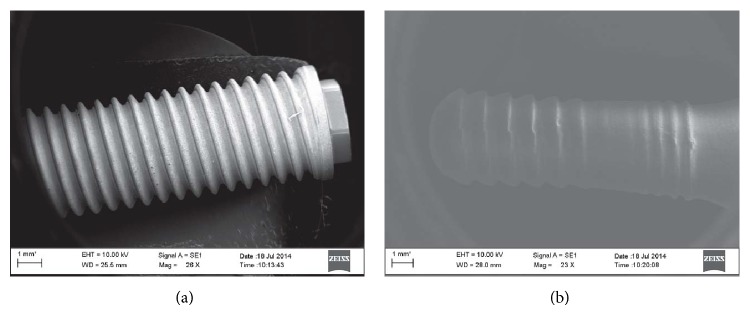
Implant geometry. Note: SEM images were acquired at low magnification to depict the shape of the titanium implant (a) and the alumina toughened zirconia implant (b).

The implant morphology is reported in Figure 1 for titanium and ceramic implants, respectively. As for the titanium implant (Figure 1(a)), only the fixture was considered for the analysis. Its geometry is represented by a set of threads, identical in each part of the screw and placed at the same distance from each other. Regarding the ceramic implant (Figure 1(b)), as it is a one piece, fixture and abutment could not be separated. The intrabony screw shows a series of threads, similar to that observed for titanium implant, in the upper part (the one closer to the abutment), while some threads having a “cup profile” are present in the apical part. Such a geometry was realized with the purpose to favor the osseointegration. Indeed, during the implant placement, the geometry of the threads does not allow a complete flowing of the removed bone. It is supposed that the residual bone could act as nucleation center for the following bone growth, so that the osseointegration may be promoted.

**Figure 2:**
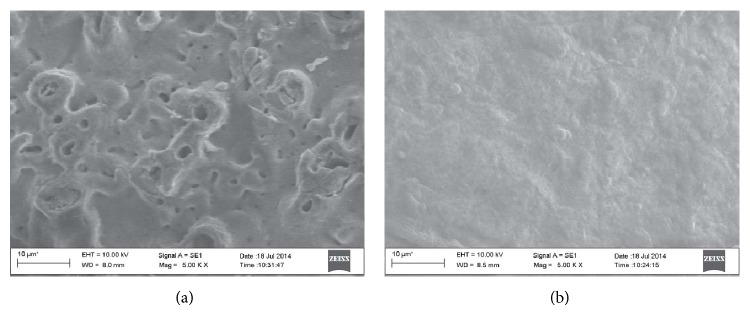
Implant surface morphology. Note: SEM images were acquired at high magnification to depict the surface morphology of the titanium implant (a) and the alumina toughened zirconia implant (b).

**Figure 3:**
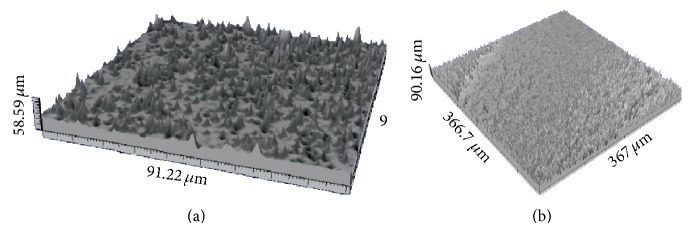
Implant topography. Note: Surface roughness of titanium (a) and alumina toughened zirconia (b) implants was determined and graphically portrayed.

A detail of the implant surfaces is reported in Figure 2. The titanium exhibits some pores along the whole surface, while the ceramic appears fully dense. The topography of the implant surface is shown in Figure 3 (Titanium, (a); ATZ, (b)). Sa values are 3.4 *μ*m and 5.4 *μ*m for titanium and ATZ implants, respectively. 

### 3.2. Mechanical Tests

As reported in the experimental section, the static test has the aim to set the conditions for dynamic tests. The results (Table 1) indicate a quite high variability of the load to failure, typical for brittle materials like ceramics. Indeed, ceramic materials generally fail because of the presence of some defects, like pores, inclusions, microcracks, and combinations. This kind of fracture mechanism leads to a large distribution of the strength, considerably higher than that of ductile materials, as metals and their alloys. An index of the variability of the strengths is represented by the Weibull Modulus: the lower the modulus, the higher the distribution. As for oxidic materials such as alumina and zirconia, Weibull modulus is about 10, while for ductile materials it is one order of magnitude higher [40]. The results of dynamic tests are reported in Table 2. All the three samples survived after five million cycles of fatigue solicitations.Therefore ATZ implants were suited to undergo further* in vivo* experiments as they met the UNI EN ISO 14801:2008 standards that are mandatory for allowing the human use.

### 3.3. Bone Markers

**Figure 4:**
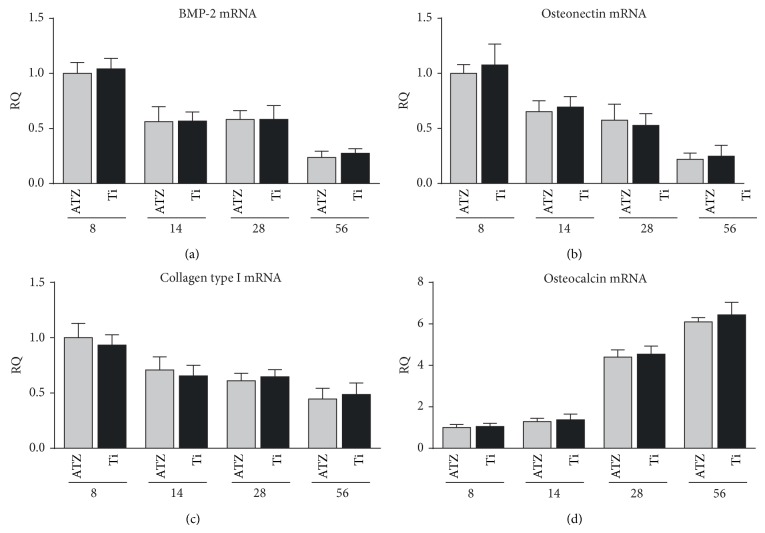
Expression of the osteogenic differentiation markers. Quantitative real-time polymerase chain reaction (RT-PCR) analysis of BMP-2 (a), osteonectin (b), collagen type I (c), and osteocalcin (d) transcript level (*n* = 3 for each condition for each time point). One-way analysis of variance (ANOVA) with* post hoc* Dunnett's test was used to assess statistical significance.

No significant difference could be detected between ATZ and titanium at the time points taken into consideration (8, 14, 28, and 56 days) for the bone markers observed (collagen type I, osteonectin, osteocalcin, and BMP-2) in peri-implant bone tissue (Figure 4).

### 3.4. Histological Analysis

**Figure 5:**
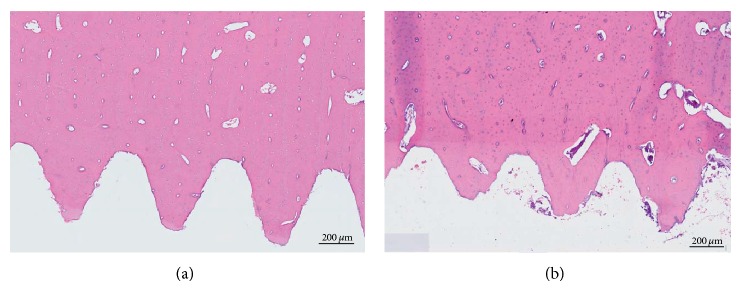
Representative histological images of cortical bone at 8 days. Note: E&E stain showing sporadic necrotic lacunae within cortical bone in proximity to the implant surface at 8 days (a) and healthy bone (b). This phenomenon occurred only at the earliest time point in a few cases for both alumina toughened zirconia and titanium implants and may be due the preparation of the implant site by drilling.

Presence of osteoid at the bone-implant interface was noted at the earliest (8 days) time point whereas little or no interfacial unmineralized matrix was seen at all other time points for both ATZ and titanium hosting bones. A minimal amount of necrotic osteocytes were noted only at 8 days in proximity to the implant surface in both groups (Figure 5(a)), at the cortical bone level, probably due to the transitory overheating during the implant site preparation. The cellular remodeling of the bone fragments was similar between the ATZ and titanium groups and activity peaked at 8 days after implantation.

**Figure 6:**
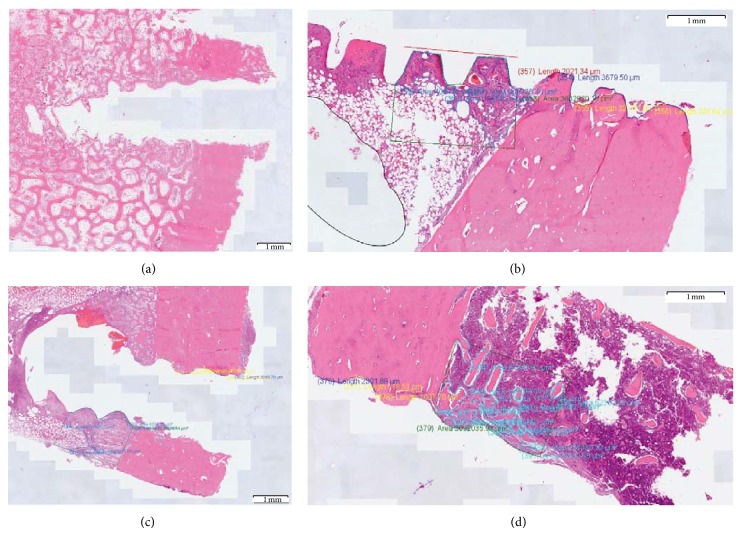
Representative histological images of DHI calculation. Representative samples of the virtual histological slides used to calculate DHI at day 56 are reported for titanium (a, b) and alumina toughened zirconia (c, d) implants, respectively, at lower (a, c) and higher (b, d) magnification.

**Figure 7:**
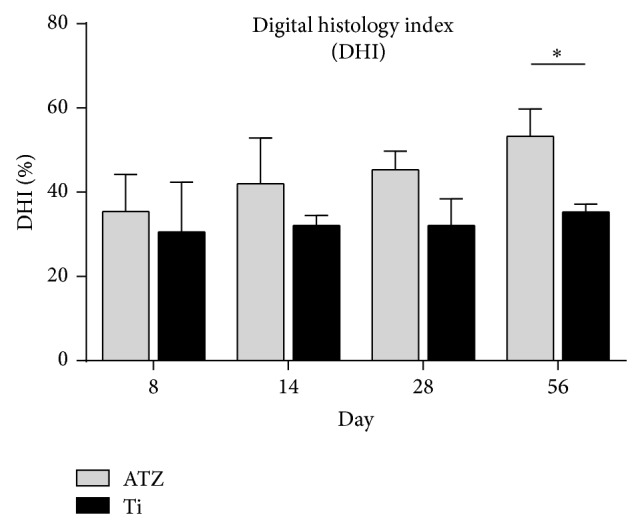
Digital Histology Index (DHI). Note: The DHI values differed in a statistically significant way (Student's *t*-test *P* < 0.001) between the alumina toughened zirconia (ATZ) and the titanium samples at day 56 (ATZ = 53.3% ± 6.5, Ti = 35.3% ± 1.9). At 28 days no significant difference could be found (ATZ = 45.4% ± 4.5, Ti = 32.1% ± 6.4).

Both treatment and control groups showed a steady increase in the overall digital histology index up to 56 days (Figure 6). The DHI values differed in a statistically significant way between the ATZ and the titanium samples at day 56 (ATZ = 53.3% ± 6.5, Ti = 35.3% ± 1.9). At the earlier time points, no significant difference could be found (at 28 days: ATZ = 45.4% ± 4.5, Ti = 32.1% ± 6.4) (Figure 7).

### 3.5. Histomorphometrical Reconstruction

The densitometric analysis of the white voxels of the medial and the apical areas of the samples concerning the 56th day is reported in Table 3. A statistical significant difference between the medial area values of ATZ and Ti was found (Student's *t*-test *P* < 0.001).

## 4. Discussion 

There is still a huge concern about the long-term durability of the Y-TZP (yttria-stabilized tetragonal zirconia polycrystal), due to the low temperature degradation (LTD) of zirconia [15–19], despite its excellent biological properties [30, 31]. In the present study, alumina-zirconia composites were chosen for manufacturing dental implants, based on their reported mechanical performances superior to those of the monolithic oxides [29, 41]. Unsurprisingly, the ATZ implants tested following the ISO standards that regulate dental implants under static and dynamic load (UNI EN ISO 14801:2008) showed satisfactory mechanical behavior. Thus, alumina toughened zirconia appears as a viable alternative to yttria stabilized zirconia, as its higher resistance to crack growth is durable and may withstand the so called ageing process taking place in aqueous environment. ATZ was selected for the* in vivo* experiment portrayed in the present paper on the basis of previous data dealing with the bioactivity assessed as per Faga et al. [35]. Indeed, only with a particular hydrothermal treatment was it possible to achieve hydroxyapatite precipitation on the ATZ samples, which did not occur, for instance, on the ZTA specimens [35].

At the end of the manufacturing process, the ATZ implants were analysed by scanning electron microscopy and submitted to profilometry for roughness evaluation, before the placement into the recipient animals, adopting a swine model previously described [42]. As a control, an anodized titanium oxide layer (TiUnite) containing anatase and rutile and endowed with a moderately rough porous surface topography was selected [43]. The ATZ implants showed an average roughness (Sa = 5.4 *μ*m) higher than that of the titanium implants (Sa = 3.4 *μ*m). Topography and surface roughness are known to positively affect the healing process [44–46]. Indeed, increasing the level of roughness ameliorates osseointegration [47], as it was acknowledged in a 2009 consensus statement [48].

Biomolecular, histological and histomorphometrical analyses were used to examine the differences in the healing and remodeling processes between the two different implant materials. Four different time points were evaluated. Representative of the early phase of healing were 8 and 14 days, while intermediate and mature bone healing were reasonably foreseen at 28 and 56 days, respectively, based on earlier experiments [49]. Interestingly, hydrothermally treated ATZ implants showed a statistically significant higher digital histology index than the titanium implants at 56 days, which is an unprecedented finding, to the authors' knowledge. Consistently, the 3D image analysis used to quantify the peri-implant bone at 56 days indicated the presence of a bone matrix denser along the ATZ than the titanium implants, particularly in the medial area.

Although ATZ is able to elicit a satisfying biological response* in vitro*, even in absence of modifications and when roughness is excluded by mirror polishing [50], no significant difference between the anodized titanium surface and the hydrothermally treated ATZ surface was detected at the mRNA level. These data could appear contradictory. However, the expression level of the investigated osteogenic markers has been normalized between the two different conditions, not taking into account the differences in the total number of cells that are effectively recruited by the two materials. Indeed, the amount of cells in the peri-implant area may differ greatly between ATZ and titanium, even if the osteogenic gene expression profile was similar in the bone forming cells growing along either ATZ or titanium. Hence, DHI was not merely dependent on the gene expression, but it could rather be affected by other surface characteristics more active on cell recruitment and proliferation than on osteoinductive features.

Based on this study, further investigations are needed before recommending the clinical use in humans, although the tested material proved to be a promising candidate among the possible ceramic dental implants.

## Figures and Tables

**Table 1:** Static mechanical tests.

	*F*_Max_ (N)
A-01	675.6
A-02	912.03
A-03	737.3

**Table 2:** Dynamic mechanical tests.

	A-04	A-05	A-06
Flexural moment (Nmm)			
*M*_mean_	586.7	566.2	567.1
*M*_dyn_	480.0	463.3	464.0
*M*_max_	1066.8	1029.5	1031.0
*M*_min_	106.7	102.9	103.1
Compression force (N)			
*F*_mean_	95.4	95.7	95.5
*F*_dyn_	78.0	78.3	79.0
*F*_max_	173.4	174.1	175.5
*F*_min_	17.3	17.4	17.5

**Table 3:** Histomorphometrical reconstruction: percentage of medullar bone density showed as mean ± standard deviation.

Voxel density	Medial area	Apical area
Alumina toughened zirconia	52.4 ± 2.6%	24 ± 4.2%
Titanium	44.2 ± 3.1%	19 ± 4.5%
